# Personal protective eyewear usage among industrial workers in small-scale enterprises

**DOI:** 10.1186/s40621-020-00280-z

**Published:** 2020-09-22

**Authors:** Tahra AlMahmoud, Ismail Elkonaisi, Michal Grivna, Fikri M. Abu-Zidan

**Affiliations:** 1 Department of Surgery, College of Medicine and Health Sciences, United Arab Emirates University, Khalifa Bin Zayed Street, P.O. Box: 17666, Al-Ain, United Arab Emirates; 2 Zayed Center for Health Sciences, United Arab Emirates University, Al-Ain, UAE; 3 Institute of Public Health, College of Medicine and Health Sciences, United Arab Emirates University, Khalifa Bin Zayed Street, P.O. Box: 17666, Al-Ain, UAE

**Keywords:** Eye injuries, Perception, Personal protective eyewear

## Abstract

**Background:**

Work-related eye injury causes significant vision loss. Most of these injuries are preventable with appropriate eye safety practices. We aimed to study industrial workers’ perceptions of Personal Protective Eyewear (PPE) and its usage in a high income developing country.

**Methods:**

A field-based cross-sectional study in small-scale industrial entities was performed in Al-Ain City, UAE during the period of October 2018 to June 2019. Five hundred workers completed a pretested structured questionnaire. Data on demographics, occupational history, work hazard awareness, and PPE usage at their work place were collected.

**Results:**

The workers were experienced, with a median of 15 years in practice. The majority (80%) learned their work skills through apprenticeship (i.e., on-the-job) training. Most (85%) were involved with activities presenting eye injury risk, and were highly aware of this. None of the workers used safety goggles or glasses all the time for activities that need PPE usage. Five percent never used PPE in the workplace. The main reason for not using PPE was the work demands (95%) and poor vision through the lenses (75%). Young age and less work experience were associated with less PPE usage (*P* < 0.0001). Wearing prescription spectacles had a positive correlation with usage of safety goggles (*P* = 0.005) and a negative correlation with welding helmet usage (*P* < 0.0001).

**Conclusions:**

There was a high level of awareness about the value of PPE in the workplace which was not translated into real practice. Educational programs promoting eye safety practices and proper PPE usage should be adopted by workers in small-scale industrial settings.

## Introduction

Ocular injury is a common cause of blindness worldwide (Pizzarello 1998; Lombardi et al. 2005; Fea et al. 2008). Around 60% of these injuries occur in the workplace (AlMahmoud et al. 2019a; Fea et al. 2008). Personal Protective Eyewear (PPE) is highly effective in preventing eye injury if appropriate selections are made available and effectively used (Lipscomb 2000; Mancini et al. 2005; Forst et al. 2006; Zgambo 2015). About 60% of work-related eye injury is related either to the lack of usage or to the wrong choice of PPE at the time of injury (Lombardi et al. 2009).

Over the past two decades, the United Arab Emirates (UAE) has dramatically increased construction and manufacturing activities that rely on welders and carpenters. Al-Ain City is located in the Al-Ain region of the Emirate of Abu Dhabi. The UAE Labour Act, Federal Law 8 (1980) and its amendments (1982) mandate regulations that promote workplace safety and affirm the enterprise’s obligation to protecting employees’ health and safety. This includes PPE supply in the workplace (Federal Law No 8 2007). Abu Dhabi Occupational Safety and Health Center (OSHAD) was established in 2010 to ensure implementation of occupational safety and health systems in the workplace (Abu Dhabi Occupational Safety and Health Center (OSHAD) n.d.). The OSHAD System Framework (SF) is a management tool that integrates Occupational Health and Safety management components of a business into one coherent system (Abu Dhabi Occupational Safety and Health Center (OSHAD) n.d.). In 2019 the Abu Dhabi Public Health Center (ADPHC) was established to maintain the health of the population and the safety of workers through the promotion of public health and preventive health concepts. ADPHC is now the independent legal body which manages the OSHAD SF (Khalifa bin Zayed issues law establishing Abu Dhabi Public Health Centre n.d.; General Secretrariate of the Executive Council 2019). The Department of Municipalities and Transport of Al Ain City Municipality is the authority that ensures that small-scale industrial workshops comply with the health requirements through legal audits and inspections (Department of Municipal Affairs and Transport, Al Ain City Municipality, Municipal Infrastructure and Assets Sector, Public Health Department 2018).

There is limited information available regarding work-related eye hazards, level of awareness and utilization of PPE among workers in small-scale industrial settings in developing countries. We recently reported the epidemiology of eye injury necessitating surgery in our setting (AlMahmoud et al. 2019a). This study covered hospitalized patients and it represented the tip of an iceberg of preventable eye injury (AlMahmoud et al. 2019a; AlMahmoud et al. 2000a; AlMahmoud et al. 2019b). Further, we have also shown a high incidence of eye injury and low usage of safety goggles among workers at small-scale industrial enterprises (AlMahmoud et al. 2020b). We therefore resolved to conduct an observational study in the workplace to directly identify risk factors for eye injury. We aimed to study the industrial workers’ perceptions of PPE and its usage in a high-income developing country so as to develop recommendations about eye injury prevention in such a setting.

## Subjects and methods

### Ethics statement

This research was approved by the Social Sciences Ethics Committee of UAE University (ERS_2017_5631). The Department of Economic Development approved the survey. Informed consent was obtained from both the workshop managers and individual workers who participated in the study.

### Study protocol

The research protocol was developed and pretested by the research team. The survey covered two separate domains related to eye injuries at small-scale industrial enterprises (AlMahmoud et al. 2020b). The first domain includes detailed question items on workers’ perceptions of PPE. The second domain has focused question items related to eye injuries and their risk factors among workers at small-scale industrial enterprises. This survey is included as an appendix to a recently published article, but the overlap between these two papers is minimal (AlMahmoud et al. 2020b). All components of the pretested structured interview survey focusing on demographic data, work experience, perception of occupational eye hazards, awareness of PPE and impediments to its usage in Al-Ain City, UAE are presented in this study. Items on workers’ perception of eye injury risk were rated on a scale of 0 ‘no harm’ to 10 ‘severe harm’. Workers’ use of personal protective eyewear (PPE) in the last week was classed as ‘always’, ‘often’, ‘sometimes’, and ‘never’. A list of options was presented for reasons for not using PPE.

### Sample size and sampling

This study was conducted among workers in small-scale industrial enterprises in Al-Ain City. The population of Al-Ain was estimated to be 631,005 (Statiscs Center- Abu Dhabi (SCAD) 2019). With the aid of the Raosoft sample size calculator (Raosoft, Inc n.d.), 5% margin of error, 95% confidence level and 50% response distribution, the calculated sample size was 384 workers. However, we aimed for 500 participants to improve validity of results.

A list of welding and carpentry workshops registered with Al-Ain municipality was obtained. Multi-stage random sampling was performed to select the study participants. A geographic map of Al-Ain City was used, and the two industrial areas were identified. The main industrial area was divided into 4 sections while the Hili industrial area was divided into 2 sections. In every section a street was randomly selected and the first small-scale industrial workshop was approached. ‘The researcher explained the protocol to each participating worker orally for clarity and to limit possible misunderstanding and then recorded the data.

### Statistical analysis

After the data were collected as hard copies, a database was designed for the study using Microsoft Access 2010 (Microsoft, Redmond, Washington USA). Data were rechecked after the data entry was completed. The data were then exported into a Microsoft Excel 2010 data sheet (Microsoft, Microsoft, Redmond, Washington USA) and coded as numbers. The Statistical Package for the Social Sciences (IBM-SPSS version 23.0, Chicago, Il, USA) was used to analyze the coded data. Data were presented as median (range) or number (%) as appropriate. Spearman Rank Correlation was used to test the correlation between two variables. A *p* value of less than 0.05 was considered as significant.

## Results

One-hundred twenty-three small-scale industrial enterprises were approached; 95 workshops agreed to participate (77% response rate). A total of 500 workers out of 518 approached in these participating workshops agreed to complete the survey (response rate of 96.5%). The median (range) age of participants was 32.5 (23–43) years. The participants were Indian (35%), Bangladeshi (30%), Egyptian (15%), Pakistani (10%), Syrian (5%), and Jordanian (5%). All participants worked for 6 days per week with a median (range) of 9 (9–10) hours per day. 30% of participants had prescription glasses whereas none used contact lenses. The median (range) of years of work experience was 15 (4–24) years. The participants had worked for a median (range) of 4 (1–12) years in UAE. 30% of participants had a diploma or higher education, 30% had secondary education, 25% had basic education and could read and write, 10% had completed elementary education, and 5% were illiterate. 80% of workers indicated that they had learned their working skills through apprenticeship training, and 95% had received occupational safety training.

Table 1 shows the tasks performed by the workers. 90% were involved in cleaning, 85% in hammering and 85% in sanding. The workers were highly aware of the risk of hot sparks and fire or explosion. There was low risk perception for bright light injury and sharp edges (Table 2). Table 3 shows the high knowledge of workers on availability of safety goggles, glasses, face shields, and welding helmets compared with filter lenses. No workers used the available safety goggles or safety glasses all the time. 5% never used PPE of any type at their workplace. 20% never used safety goggles or safety glasses. 35% never used face shields and 70% never used welding helmets (Table 4). The main reason for not using PPE was the pressure to complete the work (95%); furthermore 75% mentioned poor vision through PPE lenses, and 10% perceived no benefit for PPE usage (Table 5).

**Table 1:** Occupational tasks performed by workers (*n* = 500; Al-Ain industrial areas, November 2018–June 2019)

Type of work	Number (%)
Standing/observing/assisting	450 (90%)
Cleaning	450 (90%)
Hammering	425 (85%)
Sanding	425 (85%)
Manual handling	350 (70%)
Drilling	325 (65%)
Chipping	300 (60%)
Gas welding	275 (55%)
Arc welding (electric welding)	275 (55%)
Grinding	275 (55%)
Power sawing	250 (50%)
Painting	100 (20%)
Hand sawing	50 (10%)
Chiseling	25 (5%)

Data are presented as number (%)

**Table 2:** Workers’ awareness of seriousness of the following incidents as potential causes of eye injury or harm (scale 0–10)

Type of work	Median (range)
High temperatures/hot sparks injury	8 (4–10)
Fire or explosion	8 (2–9)
Welding fumes and gases injury	7 (0–9)
Bright light injury (UV light emitted during welding)	4.5 (1–8)
Sharp edges/ injury by metal objects	2 (0–6)
Hot tea injury	0 (0–0)
Vibration injury	0 (0–0)

Data are presented as median (range)

**Table 3:** Workers’ awareness of availability of personal protective eyewear in the workplace

PPE	Available	Not available	Unknown
Safety goggles	500 (100%)	0 (0%)	0 (0%)
Safety glasses	500 (100%)	0 (0%)	0 (0%)
Face shield	500 (100%)	0 (0%)	0 (0%)
Welding helmet	400 (80%)	25 (5%)	75 (15%)
Filter lenses	25 (5%)	25 (5%)	450 (90%)

Data are presented as number (%)

**Table 4:** Frequency of workers’ use of personal protective eyewear at workplace in the last week

PPE	Always	Often	Sometimes	Never
Safety goggles	0 (0%)	125 (25%)	275 (55%)	100 (20%)
Safety glasses	0 (0%)	125 (25%)	275 (55%)	100 (20%)
Face shield	175 (35%)	100 (20%)	50 (10%)	175 (35%)
Welding helmet	0 (0%)	25 (5%)	125 (25%)	350 (70%)
Filter lenses	0 (0%)	0 (0%)	0 (0%)	500 (100%)

Data are presented as number (%)

**Table 5:** Reasons for not using personal protective eyewear in the workplace

Reason	Number (%)
Quickly finish the job	475 (95%)
Cannot see clearly	375 (75%)
Due to hot weather	275 (55%)
Not comfortable when using PPE	250 (50%)
No benefit of using PPE	50 (10%)

Data are presented as number (%)

Young age and less work experience were both associated with less PPE usage (*P* < 0.0001). Wearing prescription spectacles had a positive correlation with wearing safety goggles (*P* = 0.005, rho 0.13) and a negative correlation with using welding helmets (*P* < 0.0001, rho − 0.42) (Table 6).

**Table 6:** Correlations between different variables and workers’ usage of personal protective eyewear (*n* = 500)

Variable		Safety goggles	Safety glasses	Face shield	Welding helmet
Age	p	0.003	0.24	< 0.0001	< 0.0001
	rho	0.13	−0.05	0.72	0.2
Education level	p	0.69	0.69	0.28	0.07
	rho	0.02	0.02	0.05	0.08
Years of experience	p	0.003	0.45	< 0.0001	< 0.0001
	rho	0.13	−0.03	0.71	0.25
Working hours	p	0.03	0.19	0.007	< 0.0001
	rho	0.1	−0.06	0.12	0.38
Wearing prescription glasses	p	0.005	0.35	0.66	< 0.0001
	rho	0.13	−0.04	−0.02	−0.42
Apprenticeship training	p	0.42	0.42	< 0.0001	< 0.0001
	rho	0.04	0.04	−0.27	−0.19

Spearman rank

## Discussion

Our study has shown that industrial workers in small-scale enterprises in our setting are mainly young men who are aware of both the types of PPE available in the workplace and the work-related eye hazards. Discomfort was the major barrier to PPE usage. This finding has also been reported by others (Budhathoki et al. 2016; Chauhan et al. 2014; Isah and Okojie 2006). Similar to other studies (Lombardi et al. 2009; Isah and Okojie 2006; El-Zein et al. 2003), our workers were highly experienced. Nevertheless, their work experience in the UAE was short. This may reflect the high rate of turnover of workers in these jobs.

Consistent with our finding, it has been reported that majority of workers learn their skills through apprenticeship training from experienced workers (Budhathoki et al. 2016). Workers in our study received some occupational safety training. Although they were aware of the presence of PPE in the workplace, they did not use it all the time when usage was needed. Low PPE usage has been reported, ranging between 9 and 18% (Ajayi et al. 2011; Omolase and Mahmoud 2007). Furthermore, reports indicate that more than 50% of workers involved in welding and carpentry do not protect their eyes during work activities (Lipscomb et al. 1999; Voon et al. 2001; Zakrzewski et al. 2017).

A significant number of participants had prescription spectacles but none used contact lenses. This could be due to financial constraints, difficulties with cleaning, or perception of hazards. Wearing prescription spectacles had a significant positive effect on wearing safety goggles in our study. Spectacle users may have been more informed and accepting of PPE usage. On the other hand, wearing prescription spectacles had a negative effect on use of welding helmets in our study. It is possible that this might be related to visibility compromise or discomfort when using more than one device. In addition, lack of provision for prescription PPE has been noted as a potential barrier to its usage (Lombardi et al. 2009).

**Fig. 1:**
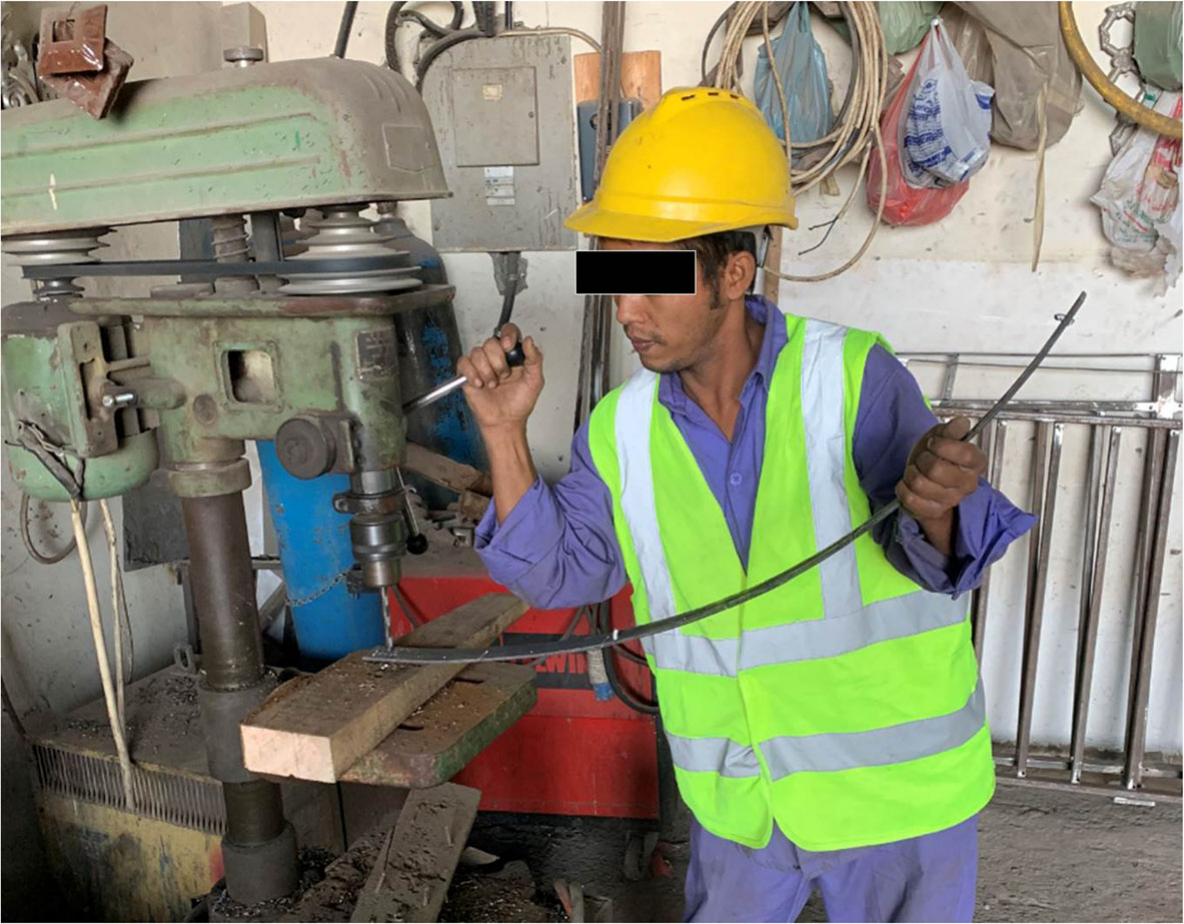
A worker at a small-scale industrial enterprise at the main industrial area of Al-Ain City drilling a hole in a metallic bar over a wood block using a large electrical drill without personal protective eyewear (The worker gave his written consent to use his picture for publication)

Eye injury occurs when the eye is not protected (Davey 1987; Kruger et al. 1990). Consistent with other findings (Zakrzewski et al. 2017; Eye Injuries n.d.), workers in our study did not use PPE all the time (Fig. 1) and 5% never used it despite being involved with high risk activities such as welding and sanding. Hammering was also a common activity for workers in our study. About 10% of medical costs of eye injuries are reported to be caused by hammering (Lipscomb et al. 1999), and are associated with low use of PPE (Fong and Taouk 1995). Other factors that contribute to eye injuries are using the wrong type of PPE and poor fit (Lombardi et al. 2009; Sukati 2014). Several types of PPE are available, and when worn and fitted properly they are highly effective in preventing the impact and potentially reducing the severity when injury occurs (Lipscomb 2000; Mancini et al. 2005; Forst et al. 2006).

Factors that influence workers’ use of PPE include discomfort, lack of fit, fogging, scratching, lack of safety training, or misunderstanding (Lombardi et al. 2005; Lipscomb 2000; Forst et al. 2006; Lipscomb et al. 1999; Winder et al. 1998; Eze et al. 2015). The majority of our workers prioritized job completion over eye protection. Furthermore, poor visibility, discomfort, and hot weather discouraged workers from using PPE in our setting. Offering workers PPE that is tailored to the local climate and tasks, including anti-fog coating, might enhance its usage (Ademola-Popoola et al. 2005; Crebolder and Sloan 2004; Earle-Richardson et al. 2014). Furthermore, apprenticeship training was negatively associated with the usage of face shields and welding helmets in our study. Workers may be following the behavior of their trainers (Lipscomb et al. 1999).

We reported earlier a high percentage of eye injury incidents among workers at small-scale industrial enterprises (AlMahmoud et al. 2020b). Prevention of such injuries may be challenging as workers, report that safety glasses are uncomfortable, increase the difficulty of work, and reduce productivity. This is of concern in the hot climate and consequent perspiration and fogging of lenses could reduce visibility through PPE. If PPE is to be accepted by workers it should be comfortable and not limit clear vision (Ademola-Popoola et al. 2005). Earle-Richardson suggested offering workers a range of eyewear and tailoring offerings to the local climate and tasks (Earle-Richardson et al. 2014). Further strategies that may lead to reductions in the burden of eye injuries by increasing the use of protective eyewear in our country could be formulated based on literature review and local systems for labour regulation and health education. Multicomponent interventions including development of appropriate educational material, workshops, and local newspaper articles containing expert advice on the subject could be adopted. Another strategy could be focus groups to collect qualitative data on behaviors, opinions, or exposures (Krueger and Casey 2014; Salazar et al. 1999; Bender and Ewbank 1994). Focus groups can also contribute to the identification of safety hazards and job risks and to informing the development and/or implementation of interventions (Salazar et al. 1999; Simpson et al. 2002; Gillen et al. 2004). These measures should be reinforced by law through a program unannounced official inspections, as such a strategy results in long term reduction in eye injuries (Mancini et al. 2005).

### Limitations

We have to acknowledge that our study has certain limitations. *First,* our study was only in Al-Ain City, hence may not reflect the situation in the whole UAE. *Second,* recall bias might have under or overestimated the results. *Finally,* where welding is concerned we were unable to quantify the time spent on this activity, as workers were involved in several activities and did welding only intermittently.

## Conclusions

Our study has shown that there is a high level of awareness of the value of PPE in the workplace which is not translated into real practice. Eye protection among workers in small-scale industrial enterprises is important. Preventive and educational strategies should be adopted to address appropriate eye protection, comfort, visibility, and specific criteria for prescription spectacles.

## Data Availability

All data generated or analyzed for this article are included in this published article.

## Abbreviation

PPE: Personal Protective Eyewear

## References

[CR1] Abu Dhabi Occupational Safety and Health Center (OSHAD). n.d. https://www.oshad.ae/en/Pages/OshadSystemViewAll.aspx. (Accessed 8 May 2020).

[CR2] Ademola-Popoola DS, Akande T, Ayanniyi A (2005). Ocular health status and Practises among the Workers of a Steel Rolling Mill in Nigeria. Cent Eur J Occup Environ Med.

[CR3] Ajayi IA, Adeoye AO, Bekibele CO (2011). Awareness and utilization of protective eye device among welders in a southwestern Nigeria community. Ann Afr Med.

[CR4] AlMahmoud T, Al Hadhrami SM, Elhanan M (2019). Epidemiology of eye injuries in a high-income developing country: an observational study. Medicine (Baltimore).

[CR5] AlMahmoud T, Elhanan M, Elshamsy MH (2019). Management of infective corneal ulcers in a high-income developing country. Medicine (Baltimore).

[CR6] AlMahmoud T, Elhanan M, Abu-Zidan Fikri M. Eye injuries caused by date palm thorns and leaves. Saudi J Ophthalmol. 2020a. 10.1016/j.sjopt.2020.04.003. 10.4103/1319-4534.301296 PMC7849857 33542981

[CR7] AlMahmoud T, Elkonaisi I, Grivna M, et al. Eye Injuries and Related Risk Factors among Workers in Small-scale Industrial Enterprises. Ophthalmic Epidemiol. 2020:1–7. 10.1080/09286586.2020.1770302. 10.1080/09286586.2020.1770302 32475211

[CR8] Bender DE, Ewbank D (1994). The focus group as a tool for health research: issues in design and analysis. Health Transit Rev Cult Soc Behav Determinants Health.

[CR9] Budhathoki SS, Singh SB, Niraula SR (2016). Morbidity patterns among the welders of eastern Nepal: a cross-sectional study. Ann Occup Environ Med.

[CR10] Chauhan A, Anand T, Kishore J (2014). Occupational hazard exposure and general health profile of welders in rural Delhi. Indian J Occup Environ Med.

[CR11] Crebolder JM, Sloan RB (2004). Determining the effects of eyewear fogging on visual task performance. Appl Ergon.

[CR12] Davey JB (1987). Industrial eye protection. Ann Occup Hyg.

[CR13] Department of Municipal Affairs and Transport, Al Ain City Municipality, Municipal Infrastructure and Assets Sector, Public Health Department, 2018. Public health requirements for commercial activities, UAE, Issue No. 4.

[CR14] Earle-Richardson G, Wyckoff L, Carrasquillo M (2014). Evaluation of a community-based participatory farmworker eye health intervention in the “black dirt” region of New York state. Am J Ind Med.

[CR15] El-Zein M, Malo J-L, Infante-Rivard C (2003). Prevalence and association of welding related systemic and respiratory symptoms in welders. Occup Environ Med.

[CR16] Eye Injuries. SCRIBD. n.d. https://www.scribd.com/document/237633499/Eye-Injuries. (Accessed 15 Nov 2019).

[CR17] Eze BI, Okoye O, Aguwa EN (2015). Awareness and utilization of welders’ personal protective eye devices and associated factors: findings and lessons from a Nigerian population. Workplace Health Saf.

[CR18] Fea A, Bosone A, Rolle T (2008). Eye injuries in an Italian urban population: report of 10,620 cases admitted to an eye emergency department in Torino. Graefes Arch Clin Exp Ophthalmol Albrecht Von Graefes Arch Klin Exp Ophthalmol.

[CR19] Federal Law No 8, For 1980. UAE Labour Law. 2007. https://www.moid.gov.ae/Laws/UAE_Labour_Law.pdf. (Accessed 28 Dec 2019).

[CR20] Fong LP, Taouk Y (1995). The role of eye protection in work-related eye injuries. Aust N Z J Ophthalmol.

[CR21] Forst L, Noth IM, Lacey S (2006). Barriers and benefits of protective eyewear use by Latino farm workers. J Agromedicine.

[CR22] General Secretrariate of the Executive Council. 2019. https://www.ecouncil.ae/ar/Official-Gazette/Documents/Arabic-2019/4Arabic2019.pdf.

[CR23] Gillen M, Kools S, McCall C (2004). Construction managers’ perceptions of construction safety practices in small and large firms: a qualitative investigation. Work Read Mass.

[CR24] Isah EC, Okojie OH (2006). Occupational health problems of welders in Benin City, Nigeria. J Med Biomed Res.

[CR25] Khalifa bin Zayed issues law establishing Abu Dhabi Public Health Centre n.d. wam. http://wam.ae/en/details/1395302763569. (Accessed 7 May 2020).

[CR26] Krueger RA, Casey MA. Focus groups: a practical guide for applied research: 5th edition. California: SAGE Publications, inc.; 2014.

[CR27] Kruger RA, Higgins J, Rashford S (1990). Emergency eye injuries. Aust Fam Physician.

[CR28] Lipscomb HJ (2000). Effectiveness of interventions to prevent work-related eye injuries. Am J Prev Med.

[CR29] Lipscomb HJ, Dement JM, McDougall V (1999). Work-related eye injuries among union carpenters. Appl Occup Environ Hyg.

[CR30] Lombardi DA, Pannala R, Sorock GS (2005). Welding related occupational eye injuries: a narrative analysis. Inj Prev J Int Soc Child Adolesc Inj Prev.

[CR31] Lombardi DA, Verma SK, Brennan MJ (2009). Factors influencing worker use of personal protective eyewear. Accid Anal Prev.

[CR32] Mancini G, Baldasseroni A, Laffi G (2005). Prevention of work related eye injuries: long term assessment of the effectiveness of a multicomponent intervention among metal workers. Occup Environ Med.

[CR33] Omolase CO, Mahmoud AO (2007). The welders protective goggles: an evaluation of its appreciation. Niger J Surg Sci.

[CR34] Pizzarello LD (1998). Ocular trauma: time for action. Ophthalmic Epidemiol.

[CR35] Raosoft, Inc. Sample Size Calculator. n.d. http://www.raosoft.com/samplesize.html. (Accessed 6 Oct 2018).

[CR36] Salazar MK, Takaro TK, Connon C (1999). A description of factors affecting hazardous waste workers’ use of respiratory protective equipment. Appl Occup Environ Hyg.

[CR37] Simpson EM, Moll EK, Kassam-Adams N (2002). Barriers to booster seat use and strategies to increase their use. Pediatrics.

[CR38] Statiscs Center- Abu Dhabi (SCAD) (2019). Publication- Statistical year book of Abu Dhabi.

[CR39] Sukati VN (2014). Workplace eye injuries: a literature review. Occup Health South Afr.

[CR40] Voon LW, See J, Wong TY (2001). The epidemiology of ocular trauma in Singapore: perspective from the emergency service of a large tertiary hospital. Eye Lond Engl.

[CR41] Winder C, Dingsdag D, Dain S (1998). Development of training programs for eye safety in the NSW coal mining industry [Extracts of this paper were presented at the ANZAOHSE. Conference (1998: Brisbane)]. J Occup Health Saf Aust N Z.

[CR42] Zakrzewski H, Chung H, Sanders E (2017). Evaluation of occupational ocular trauma: are we doing enough to promote eye safety in the workplace?. Can J Ophthalmol.

[CR43] Zgambo J (2015). Occupational hazards and use of personal protective equipment among small scale welders in Lusaka, Zambia.

